# A systematic review of preoperative radiological factors associated with the development of low anterior resection syndrome (LARS)

**DOI:** 10.1007/s13304-025-02184-3

**Published:** 2025-04-05

**Authors:** Gianpiero Gravante, Veronica De Simone, Gaetano Gallo, Giuseppe Currò

**Affiliations:** 1 Department of General Surgery, Azienda Sanitaria Locale ASL Lecce, Casarano, Italy; 2 Proctology and Pelvic Floor Surgery Unit, Ospedale Isola Tiberina-Gemelli Isola, 00186 Rome, Italy; 3 Department of Surgery, “Sapienza” University of Rome, Rome, Italy; 4 General Surgery Unit, Department of Health Sciences, University “Magna Graecia” Medical School, Catanzaro, Italy

**Keywords:** Low anterior resection syndrome, Pelvic floor diseases, Radiology, Magnetic resonance imaging, Ultrasound

## Abstract

The aim of this systematic review is to summarise the available evidence for radiological changes associated with postoperative low anterior resection syndrome (LARS). A literature search was undertaken for all studies focusing on preoperative radiological predictors of postoperative LARS. Articles were selected from MEDLINE, EMBASE and the Cochrane Central Register of Controlled Trials (CENTRAL) databases up to October 2024. Eighty-four articles were screened: eighty-one were excluded and three were included in the analysis. All included studies focused on preoperative Magnetic Resonance Imaging (MRI) already performed as part of the oncological assessments, no study examined ultrasound or defecography. Authors retrospectively selected patients that underwent LAR, screened them with the LARS score, and reviewed preoperative MRI images with specific softwares in order to find radiological characteristics associated with LARS. Results showed that particular anatomical characteristics were present in patients that subsequently developed major LARS: the volume of the pubococcygeal + iliococcygeus muscles in 27 LARS patients out of 46 LAR (odds ratio—OR 14.7, 95% CI 1.7–128.3; *p* = 0.02), the thickness of the anorectal joint in 136 LARS out of 255 LAR preceded by neoadjuvant chemoradiotherapy (OR 0.653, 95% CI 0.565–0.756; *p* = 0.001) and the mesorectal/pelvic volumes in 135 LARS out of 236 LAR (Cox Regression analysis, *p* = 0.0017 and *p* = 0.0001 respectively). Pelvic floor musculature is a factor, among the others, that contributes to LARS. Future prospective studies need to validate these retrospective results, further delineate its influence, and investigate the potential contribution of other radiologic investigations (ultrasound and defecography) in this setting.

## Introduction

Low anterior resection syndrome (LARS) is a relatively recent entity, defined as a functional impairment in defecation following the surgical removal of the rectum (low anterior resection—LAR) that significantly affects the patient’s quality of life [1]. The syndrome manifests with different symptoms, mostly fragmented defecation, incontinence, obstructed or painful defecation and urgency, that result in a functional and social impairment of the daily activities [2]. As the definition of LARS relies on a combination of various symptoms, each with a variable degree of expression, it is difficult to establish its true prevalence. Although a broad and more inclusive definition would diagnose LARS in 90% of postoperative patients (almost all present alterations in bowel habits following low anterior resection) [1], a more reliable estimation sets a prevalence of 41–42% [3].

The impact of LARS on the patient’s life is significant and affects physical, cognitive and social functions [4]. The effects on the patient’s life, as evaluated by adequate functional scores [5], are directly proportional to the LARS severity [6]. Major LARS, the worse manifestation of the syndrome, is present in 14–69% of patients and is associated with the worse scores [4, 6–9]. LARS is, by definition, the consequence of LAR even if similar symptoms can also be present in a population which still has not, or never will, undergo rectal surgery [10]. In these patients anatomical and functional modifications, already present before surgery, act as combined etiological factors in the postoperative occurrence of symptoms and LAR is an aggravating factor, not the only cause, on an already predisposed background [10].

In the last decade numerous studies have focused on clinical factors associated with the postoperative development of LARS. Prediction of this particularly invalidating syndrome (especially in the major forms) could help during the informed consent process as well as after surgery to select those patients particularly at risk, where specific postoperative strategies would be useful to prevent or treat the syndrome early [11]. Despite numerous clinical factors have been reported so far, only few studies have focused on radiological predictors. The purpose of this systematic review is to report results of those studies in which radiological factors have been associated with the postoperative development of LARS.

## Materials and methods

A review protocol was registered (registration number: CRD42025630096) a priori in the International Prospective Register of Systematic Reviews (PROSPERO¸ https://www.crd.york.ac.uk/PROSPERO/view/CRD42025630096). The authors developed the protocol for review, detailing pre-specified methods of analysis and eligibility of the studies, in line with the 2020 Preferred Reporting Items for Systematic Reviews and Meta-analyses (PRISMA) guidance [12]. Inclusion criteria consisted in studies focusing on LAR patients (both oncological and non-oncological) that underwent preoperative imaging in which radiological factors were found associated with postoperative LARS. Excluded were those studies involving interventions other than LAR, where imaging was conducted after surgery (therefore not useful to predict the postoperative insurgence of LARS), or where no mention was done of eventual radiological factors associated with postoperative LARS.

All types of studies (retrospective, prospective, observational studies, clinical trials) were included. Articles were selected from MEDLINE, EMBASE and the Cochrane Central Register of Controlled Trials (CENTRAL) databases between 2000 and October 2024. The search strategy was conducted using two different sets of key words, one for LARS, one for radiology. Key words used for LARS were LARS and Low Anterior Resection Syndrome. Key words used for radiology were Magnetic Resonance Imaging (MRI), Ultrasound (US)—including “pelvic floor US”, “endoanal US”, “endovaginal US”—and defecography. No language restrictions were employed.

**Fig. 1:**
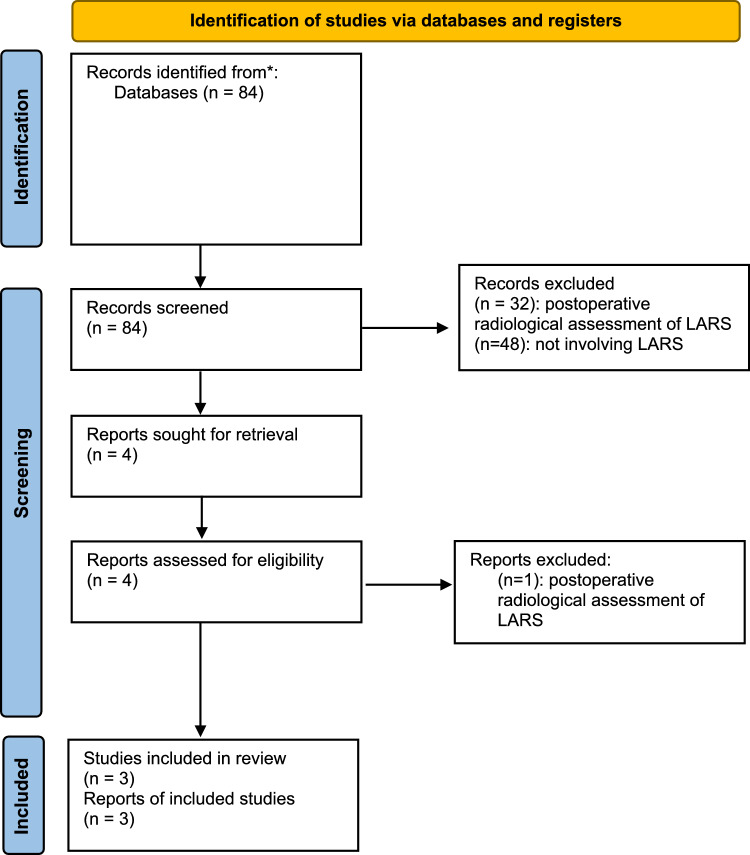
PRISMA flow-chart showing the retrieval, analysis and selection process of articles included in the systematic review

Potentially relevant articles were identified by the title and the abstract and full papers were obtained and assessed in detail (Fig. 1) by two independent reviewers (GGr; GGa) prior to their inclusion in the review. The reference list for each article (including copies of previously published reviews on the topic) was also screened to identify further relevant publications, which were obtained and assessed. Finally, the Current Controlled Trials (http://www.controlled-trials.com) database was also screened for randomized trials currently ongoing. Discrepancies were resolved by discussion or consultation with a third author (VDS).

Data regarding the study characteristics, preoperative methods of radiological assessment, number of patients undergoing LAR, those developing LARS and its severity, the radiological variables investigated and those associated with LARS (including the level of statistical association) were extracted and reported. Data analysis was carried out independently by one researcher (GGr). The methodological quality of the included studies was assessed with the Newcastle–Ottawa scale [13]. Results are presented in three different paragraphs: epidemiology and pathogenesis, clinical assessment of LARS symptoms and severity, and role of radiology for the prediction of LARS.

## Results

### Relevant epidemiology and pathogenesis

The incidence of LARS is relatively high following rectal surgery, between 64% [7] and 77% [6], while its severe forms vary between 14% [9, 14] and 69% of patients [4]. These variations likely derive from the patient’s subjective interpretations of symptoms and from the personal perception on how these affect influence his quality of life. Historically, the main risk factors associated with postoperative LARS were the presence of radiotherapy (RT) and the distance of the cancer from the anal verge. RT increases the risk of LARS by 20-folds and the effects are evident even in patients not undergoing LAR (i.e. RT to treat prostate cancer), configuring the treatment in itself a definite risk factor for LARS [15, 16]. Preoperative RT acts by damaging pelvic structures, including the autonomic plexus, and the rectum itself (the submucosal plexus and rectal mucosa sensitivity) [11, 15]. Ninety per cent of patients undergoing RT experience LARS symptoms, 50% of them have their quality of life significantly affected [15]. The cancer level from the anal verge is a surrogate for the level of the anastomosis performed, which in turn is a surrogate for the amount of rectum removed. This significantly impacts the postoperative quality of life because it is directly related to the residual functions left (reservoir, compliance, perceptive properties, coordination of sphincter relaxation and propulsive movement) [17, 18]. The lower the anastomosis, the higher the incidence and severity of LARS symptoms.

Interestingly, patients that did not receive rectal surgery also may present symptoms similar to LARS in 27–38% of cases (15% with severe forms) [10, 14, 19–21]. Gender differences have been observed (female sex has higher incidences) [9, 14, 19] possibly because of gender-specific factors such as pregnancy and mode of delivery. Deep endometriosis as well may present with symptoms similar LARS in 38% of patients [22, 23], and rectal shaving for endometriosis produce them in 30% of patients [24]. Other factors such as aging, gynecological surgery, comorbidities (i.e. diabetes), and neurological medications, that normally influence the correct function of pelvic floor muscles, have all been related to symptoms similar to LARS [9]. These factors overall explain the presence of such symptoms in the population that has still not received LAR (or never will), or act as important contributing factors for the development of LARS in those patients subsequently undergoing LAR.

Numerous causes are involved in the pathogenesis of LARS, collectively classified into modifications of distal colonic motility, the anal sphincter function and dysfunctions of the neorectum [11, 25]. These contribute to the normal continence/defecation and the disruption of one or more of them produce the insurgence of LARS symptoms. The amount of disruption and the possible combinations of factors involved produces a constellation of pathophysiological changes leading to different clinical manifestations. Rectosigmoid resection disrupts the physiological “rectosigmoid brake”, a physiological mechanism that prevents overfilling of the rectum and incontinence that is normally present at the rectosigmoid junction (also called “functional sphincter”) [25, 26]. The disruption can manifest in the way of a hypoactive as well as hyperactive junction, leading to symptoms of either incontinence or impaired defecation. It also explains one of the mechanism of action of sacral neuromodulation, that normally restores the physiological brake present in the distal colon [25]. Rectal surgery may cause damage to the internal anal sphincter because of alterations of the normal anatomy (intersphincteric resections) [27], insertion of the stapler device [28], damage to the nerve supplying the sphincters (located posterolaterally to the prostate) [29]. Dysfunctions of the neorectum derive from both changes in neorectal compliance (poor reservoir function) and rectal denervation, that produces dysmotility and altered colo/rectoanal sensation [25]. These last two factors decrease propagation waves and produce periodic spastic contractions, leading to prolonged transit times in the neorectum and symptoms of fecal urgency [30].

### Clinical assessment of symptoms and severity

Over the years various attempts have tried to classify LARS manifestations according to the prevalent combination of symptoms. These are numerous and involve different aspects of defecation and continence, however two main manifestations can be recollected, namely evacuatory dysfunction (excessive straining, incomplete evacuation, more unsuccessful bowel movements, anal/vaginal digitations, regular enema use, fragmented defecation, greater than 10 min toileting) and storage dysfunction (leaking stools or flatus, use of incontinence pad, faecal urgency, use of antidiarrhoeal medication, lifestyle modifications) [31]. These two classes of symptoms correspond to two LARS phenotypes, the first with symptoms mainly related to evacuatory dysfunction and the second to symptoms of inadequate storage [31]. These phenotypes are obviously a simplification of the reality and they are usually both present, in different amounts, in the same patient. However, they have to be considered as a pragmatic starting point on the initial assessment LARS patients and the effects they have on the patient’s quality of life, in order to guide subsequent treatments. Of note, storage dysfunction symptoms seem more influent on quality of life than evacuatory dysfunction, and patients with major LARS complain most of storage problems [31].

Along with the determination of the quality of LARS symptoms it is important to score the severity of the disease. Over the years, various clinical scoring systems have been produced that aimed to classify the amount that, overall, LARS symptoms had on the patient’s quality of life [32]. The most frequently used scoring system is based on 5 symptoms (incontinence for flatus, incontinence for liquid stools, frequency, clustering, urgency) with a range (0–42) that was divided into three groups: no LARS (0–20) minor LARS (21–29), major LARS (30–42) [33].

### The role of radiology in preoperative LARS prediction

After an initial search, 84 potentially relevant articles were screened. Forty-eight articles did not provide any data on LARS and were immediately excluded. Thiry-two studies included MRI performed after surgery to investigate LARS when this was already present; these were also excluded as they did not meet the purpose of our review, which focused on the preoperative radiological prediction of LARS. Four articles were retrieved and analyzed in detail. However, one of them involved MRI performed after surgery and was therefore excluded. As a result, three articles remained suitable for the analysis (Table 1, Fig. 1) [34–36]. Based on the Ottawa–Newcastle scale, all article were of good quality (7–8/9 points): they had a well-defined patient population, an objective measurement of the exposure (the MRI), an adequately long follow-up for the outcome to occur, and adjusted for confounders through the multivariate analysis; limitations consisted in the lack of a non-exposed control group (i.e. patients without LAR), the LARS assessment of the questionnaire was self-reported (recall bias), and provided an inadequate information of patients lost during the follow-up (i.e. number, reasons; potential selection bias—Table 2). Additionally, no quantitative analysis of results was possible due to the paucity of included studies, the heterogeneity of the variables analysed and the different statistical methods used.

**Table 1:** Studies on radiological predictive factors of low anterior resection syndrome

Author	Year	Subjects (*n*)	Imaging modality	Radiological parameters investigated	Univariate analysis	Multivariate analysis
Mori al. [34]	2022	46 no RT	MRI	3-D volume calculations of pubococcygeal muscle + iliococcygeus muscle, puborectal muscle, external anal sphincter, internal anal sphincter	Sex, lateral lymph node dissection, diverting ileostomy, external anal sphincter + puborectal muscle, pubococcygeal + iliococcygeus muscle	Sex, diverting ileostomy, pubococcygeal + iliococcygeus muscle volume
Zhang et al. [35]	2022	255 RT, 72 no RT	MRI	Thickness of levator ani, anorectal joint, internal and external anal sphincter, distance between tumor’s lower edge and anorectal junction / anal verge, anal canal length, anorectal angle, distance between bilateral ischial spines	RT: sex, thickness of levator ani and of anorectal junction, distance of tumor and anorectal junction or anal verge No RT: thickness of anorectal junction, thickness of external anal sphincter, pT	RT, thickness of anorectal joint, distance between tumor and anorectal junction
Wang et al. [36]	2024	236 no RT	MRI	Tumor distance from the anal verge, tumor length, tumor diameter, tumor proportion of the intestinal wall, mesorectal infiltration depth, circumferential resection margins and tumor volume; the anorectal angle, mesorectal volume and pelvic volume	Body weight, tumor location < 7 cm from the anal verge, small mesorectal and pelvic volumes	Tumor location < 7 cm from the anal verge, small mesorectal and pelvic volumes

MRI = Magnetic Resonance Imaging. RT: radiotherapy. BMI: Body Mass Index

**Table 2:** Newcastle–Ottawa scale of included studies

	Representativeness of exposed cohort	Selection of non-exposed cohort	Ascertainment of exposure	Outcome not present at baseline	Comparability of cohorts based on design or analysis	Assessment of outcome	Follow-up long enough	Adequacy of follow-up of cohorts	Points
Mori al. [34]									7/9
Zhang et al. [35]									7/9
Wang et al. [36]									8/9

The three studies included were all retrospective and recently published [34–37]. They all adopted the same methodology: they initially classified patients that underwent through LAR into two separate subgroups according to the presence or LARS via a self-administered LARS questionnaire. For each patient numerous clinical and oncological data were collected, then they retrospectively reviewed preoperative MRI images with specific softwares to collect radiological data about variables considered potentially influencing the postoperative LARS. Finally, they performed an initial univariate analysis to screen all variables between LARS and non-LARS groups; those that resulted significant at the univariate analysis were subsequently added in the multivariate analysis.

Mori et al. in a pioneer article focused the volume of continence-related muscles (internal and external anal sphincters, levator ani—measured in its separate components) from MRI images performed for rectal cancer staging prior to surgery [34]. Excluded were those patients with documented preoperative defecation dysfunctions, that underwent RT or intersphincteric resections. Results on 46 patients showed at the univariate analysis that sex, lateral lymph node dissection, diverting ileostomy, external anal sphincter + puborectal muscle volumes, pubococcygeal + iliococcygeus muscle volumes were significant different between major LARS vs. minor or no LARS at the univariate analysis [34]. The significance was confirmed at the multivariate analysis with regards to the pubococcygeal and iliococcygeus volumes, along with sex and the presence of a diverting ileostomy [34].

The study of Zhang et al. was conducted on two separate groups, patients that received (*n* = 255) or not (*n* = 72) preoperative RT [35]. The exclusion criteria, among the others, involved patients with an intestinal stoma. The Authors calculated the thickness of muscles involved in defecation (levator ani, internal and external anal sphincters), the anorectal joint angle and thickness, the anal canal length as well as classic oncological parameters (distance between tumour’s lower edge and anorectal junction or the anal verge) [35]. A univariate analysis was conducted to screen variables among LARS vs. non-LARS subgroups, then the multivariate logistic regression analysis was conducted to screen for independent factors for predicting severe LARS. This analysis was conducted separately for patients undergoing or not preoperative RT, comparing in each subgroup patients with or without LARS. In patients with preoperative RT a lower tumor location, thinner thickness of the anorectal joint, thickness of the levator ani and shorter distance between the tumor and the anorectal angle were associated with major LARS. In patients without preoperative RT a lower tumor location, thinner thickness of the anorectal joint, of the external anal sphincter, and a shorter distance between the tumor and the anorectal angle were all associated with major LARS. The only parameter significant associated at the multivariate analysis in both groups was the distance between the tumour and the anorectal junction, however in the group undergoing preoperative RT also the thickness of the anorectal joint [35].

Wang et al. was conducted on 236 patients that did not receive preoperative RT [36]. MRI parameters consisted in this study mostly of oncological measurements as well as pelvic volume measurements without focusing specifically on pelvic floor muscles (Tab.1). The univariate analysis found significant differences bwteen LARS and non-LARS patients for the body weight, tumor distance from the anal verge, small mesorectal and pelvic volumes. The multivariate analysis showed tumor distance from the anal verge, small mesorectal and pelvic volumes as associated with the insurgence of postoperative LARS [36].

## Discussions

LAR is a well-known and established operation for the treatment of rectal cancer, with numerous cases performed annually worldwide. Modifications of bowel habits and changes in the fecal continence are frequently observed due to the complete or partial loss of the organ’s function. Considering the huge number of patients undergoing LAR yearly, and the frequency of functional bowel modifications observed afterwards, a large number of patients are affected by LARS annually with a significant impact on their quality of life.

Despite these premises it is only in recent decades that the focus of the scientific community has shifted from the cure of the disease to the cure of the person, including the overall postoperative well-being. Colorectal symptoms, initially reported as necessary side-effects of the oncologic operation, have now been formally named into a specific syndrome (LARS) and dedicated more attention [1, 2, 32]. Over the years numerous studies concentrated on ways to diagnose LARS, screen its presence, define the incidence in the LAR population, score the severity [5, 33], establish risk factors, test treatments [38–42], and finally create *ad-hoc* guidelines of management [43]. More recently, interest has also been developed in preventive strategies. These focused on “pre-habilitating” the patient to the potential postoperative LARS through pelvic floor exercises [44, 45] and dietary changes [46–48], and suggestions have been made (to be tested in future trials) that optimising timing of ileostomy closure [49, 50], and different methods of surgical reconstructions could diminish the postoperative incidence of LARS [46].

However, the discovery of similar symptoms also in the population not undergoing LAR opens new perspectives. Approximately one third of people that do not receive rectal surgery suffer of symptoms similar to LARS with higher incidences in women, old patients, those with previous gynaecological surgery, neurological comorbidities and medications affecting the neuromuscular apparatus. All these categories have in common an anatomical or functional neuromuscular impairment of their pelvic floor muscles where the consequence is the clinical presence symptoms similar to LARS. Furthermore, such risk factors might be present also in patients undergoing LAR that still do not manifest symptoms, but where LAR might be sufficient to unmask them through the production of LARS. These findings open the way to new methods of LARS screening, to detect the presence of such risk factors preoperatively and therefore start a “pre-habilitation” process.

Pelvic floor muscles can be investigated in different ways, each one evaluates an aspect of their anatomy and function. Radiological techniques, namely US, MRI, defecography, define the anatomy of muscles involved, the efficacy of the defecation, and any influencing associated pathology (i.e. pelvic floor organ prolapses) [51, 52]. Manometry help to quantify the muscular strength [53], electromyography investigates the anatomical and functional integrity of the neuromuscular pathways. All these techniques, together, share different views on the functionality of pelvic floor; their combination is necessary to assess the overall quality of defecation and continence. It is reasonable to hypothesize that they could be used to screen patients undergoing LAR for the presence of non-surgical-related risk factors that might increase the risk of postoperative LARS.

However, the analysis of the literature has produced only three studies, all of them published in the last few years and based on preoperative MRI prior to rectal cancer surgery [34–36]. Two of them were conducted on patients that did not undergo RT (the importance of this factor, compared to the others, is paramount on the insurgence of LARS) while the third separated results according to the RT status. They all investigated altogether radiological morphological factors with others oncological (i.e. the distance from the anal verge, tumour dimensions) or demographic characteristics (i.e. age, sex, Body Mass Index). In their analysis, the Authors started with a univariate assessment to screen factors potentially associated with overall LARS and major LARS, only to add in the multivariate analysis those factors that resulted significant. Of note, no study reported a power-size analysis, especially in consideration of the proportion between the number of variables inserted in the multivariate analysis and the number of patients analysed [34–36]. In two studies results suggested that some among pelvic floor muscles, namely the pubococcygeal and iliococcygeus and, in RT patients, those of the “anorectal joint” (the lower edge of the levator ani and the deep part of the external anal sphincter) were associated with LARS [34, 35].

Apart from the MRI, no other radiological or functional methodology has ever been assessed preoperatively for the prediction of LARS. The three studies presented in our systematic review used MRI, routinely performed during the preoperative workup of rectal cancer, to obtain data potentially useful to predict LARS via specific softwares. Although US and defecography are also useful in the management of LARS, they are not performed routinely in the preoperative setting. This is a significant limitation in the interpretation of the MRI findings described above, but defines important new lines of research: future studies should now explore the effectiveness of US and defecography to predict LARS and integrate results achieved with those of the MRI. This should be done in well-planned prospective trials in order to control for confounding factors that are numerous, as evident from the analysis of studies presented. Furthermore, radiology alone might miss muscular functional and not anatomical impairments that do not manifest on imaging, leading to false negatives when predicting the LARS risk. Therefore, a necessary integration should be done also with functional studies (i.e. manometry) to investigate the influence of function, and not only anatomy, on the occurrence of LARS. All these data could help overall delineate scores of risk for the development of defecation/continence modifications, thereby alerting the clinicians to which patient might need particular informed consent and prepare preoperative *ad-hoc* preventive strategies.

## Conclusions

Published studies have limitations that still do not allow definitive conclusions; however, they are the first to point out at the important role that pelvic floor muscles might have on the occurrence of postoperative LARS. This role, if confirmed, opens the way to more precise preoperative assessment in order to start preventive strategies in a peculiar high-risk subset of patients.

## Data Availability

The data that support the findings of this study are available from the corresponding author upon reasonable request.
